# Expanding the Core Foundations of Global Health Law through a Pandemic Agreement

**DOI:** 10.1017/jme.2025.10151

**Published:** 2025

**Authors:** Pedro A. Villarreal

**Affiliations:** Global Issues, German Institute for International and Security Affairs, Germany

**Keywords:** Pandemic Agreement, International Health Regulations (IHR 2005), Equity, Pathogen Access and Benefit-Sharing (PABS), World Health Organization

## Abstract

The adoption of the main text of the Pandemic Agreement at the 2025 World Health Assembly is a milestone in global health law. The adopted text makes several key contributions, but there were several missed opportunities in the negotiating process, and key roadblocks remain for the future of the Pandemic Agreement.

## Introduction

The main text of a Pandemic Agreement was adopted at the May 2025 World Health Assembly by Member States of the World Health Organization (WHO). This was hailed as a win for multilateralism and a reminder that important health decisions involving the international community of states can still be made. Key concepts such as One Health and equity, reflecting policy challenges and failures in the COVID-19 pandemic, have now been given legal definitions, offering national authorities a common framing for future pandemic prevention, preparedness, and response. Similarly, the pandemic agreement has multiple linkages to the amendments of the International Health Regulations (IHR) of 2005, adopted in 2024 and expected to enter into force for States Parties that do not opt out of them in September 2025. These linkages will contribute to a synergy between both legal instruments, yet such synergies will depend on their implementation.

At the same time, the adoption of the main text by the World Health Assembly is but the first, albeit crucial, step towards having a legally binding Pandemic Agreement. A key component remains open: the negotiation of a Pathogen Access and Benefit-Sharing (PABS) System, considered by some as crucial for the realization of equity in future pandemics. In fact, the ultimate fate of the Pandemic Agreement will hinge upon this component, with Article 31.2 making explicit that the agreement will not open for signature until the successful conclusion of an Annex on PABS.

This column addresses the key contributions of the main text of the Pandemic Agreement adopted so far, as well as several limitations and open challenges for the next stages of negotiations of an Annex on PABS. The analysis emphasizes how the adopted text of the pandemic agreement directly refers to the 2024 amendments of the IHR (2005). However, a few critical remarks are put forward concerning the gradual watering down of some provisions during negotiations and, closely related thereto, some missed opportunities. This column concludes with a few notes concerning how the adoption of the main text of the pandemic agreement sheds light upon the current geopolitical roadblocks for multilateralism under global health law.

## Key Contributions of the Pandemic Agreement

The adoption of the main text of the Pandemic Agreement by the World Health Assembly^1^ is a welcome achievement for the multilateral system in general — and the WHO in particular. The fact that WHO Member States have overwhelmingly agreed on a set of common obligations under global health law is, in itself, of added value. As one negotiator remarked, it is essential to draft a text adopted by states representatives, which is capable of offering a structured way to guide action in future pandemics.^2^ Such a set of commonly agreed-upon guidance was mostly absent when COVID-19 emerged, as the IHR (2005) did not address several key questions of global health equity.^3^

The potential future entry into force of the Pandemic Agreement would lead to the creation of a Conference of the Parties, which would oversee the Agreement’s implementation through periodic meetings. This new body would operate under the principle of “one state, one vote,” and would be linked to the States Parties Committee for the Implementation of the IHR (2005), with both having a “non-adversarial, non-punitive”^4^ role meant to offer advice rather than to coerce states. The creation of these two bodies also reaffirms the Member State-driven nature of obligations under these legal instruments, which could contribute to the implementation of those obligations as a matter of “shared sovereignty.”^5^

Furthermore, the new definition of a “pandemic emergency” under global health law is expected to serve as a bridge between the IHR (2005) and the Pandemic Agreement. According to common Article 1 of both instruments, a pandemic emergency is “a public health emergency of international concern, that is caused by a communicable disease” and which “has, or is at high risk of”: spreading widely across and within multiple states; overwhelming healthcare systems; causing socioeconomic disruption; and requiring coordinated international action that is “rapid and equitable.”^6^ The WHO Director-General will have the authority to declare a pandemic emergency, which currently affords much leeway to interpretation given how the definition, understandably, does not offer fixed quantitative criteria for determining when exactly an event is a pandemic.^7^

Notably, article 12.6 of the Pandemic Agreement directly links the operationalization of the PABS System to the declaration of a pandemic emergency under IHR (2005). According to this provision, pharmaceutical manufacturers bear obligations to grant a minimum of 10% of real-time production of “safe, quality and effective vaccines, therapeutics, and diagnostics for the pathogen causing the emergency,” which are subject to contracts previously signed by these manufacturers with the WHO. Pandemic emergency declarations by the WHO Director-General have been issued in the past, both in the case of H1N1 Influenza in 2009, and more recently for COVID-19 — albeit in the latter case with a different wording.^8^ These declarations, moreover, had concrete consequences, as they activated clauses in “dormant” contracts that triggered a specific set of obligations for their parties.^9^ Nevertheless, the inclusion of contractual clauses referring to pandemic emergency declarations by the WHO Director-General, and declarations to the effect that a pandemic emergency has ended, has been mostly optional and not an established practice until now. The direct link in the Pandemic Agreement to declarations of a pandemic emergency under the IHR (2005) will ensure that the activation through these declarations of contractual clauses in contracts signed within the PABS system will not depend upon ad hoc decisions by the parties to those contracts, but will rather be a permanent component of pandemic responses. Broadly speaking, this offers a higher degree of certainty and stability for knowing when the clauses of the PABS system will have to be implemented by participating manufacturers who sign an agreement with the WHO.

Moreover, if and when the Pandemic Agreement does enter into force in the future, it could be the first legally binding treaty to enshrine a One Health perspective. The COVID-19 pandemic exposed the limitations of the prevailing “silo mindset,”^10^ in which human and animal health and the environment are subject to entirely separate regulations, falling under the mandate of different international organizations.^11^ This silo perspective was, and still is, counterproductive to pandemic prevention, preparedness, and response, as evidence about the link between human encroachment upon the environment, and the different interactions with non-human animals, increase pandemic risks.^12^ The obligations under the One Health approach in the Pandemic Agreement do not refer to concrete actions or goals by states, falling quite short of what others have called a “deep prevention” approach.^13^ Nevertheless, the provision on One Health under the Pandemic Agreement offers a template for the future adoption of domestic policies, which can then be reported to the Conference of the Parties.^14^

The protection of the health and care workforce during pandemic emergencies is a vital component of any preparedness and response plan, as it is now clearly recognized in article 7 of the Pandemic Agreement. The future implementation of legal obligations must prioritize the protection of first responders during a pandemic, such as by offering speedy access to pandemic-related health products. There is also a commitment by states to offer a “safe and healthy” work environment to other essential workers during pandemic emergencies, although the list of which occupations will be considered “essential” rests upon national authorities.^15^ It is worth noting that this article had been agreed upon by all delegations since 2024, showing a broad political recognition of the high-risk role played by health and care personnel during pandemics. This role is all the more crucial when an effective vaccine or other medical countermeasure is not available in the initial stages of pandemic response.

Another key lesson learned from the COVID-19 pandemic is the extreme pressure upon existing medical supply chains,^16^ which are designed to guarantee the manufacturing of products to meet the demands of potential purchasers in non-emergency times. Therefore, a Global Supply Chain and Logistics Network will potentially be created under article 13 of the Pandemic Agreement. The goal of this Network is, among other things, to gather information about existing medical supply chains that will become necessary during a future pandemic, ideally to identify current and future gaps that could impede the equitable distribution of pandemic-related health goods. According to article 13.1 of the Pandemic Agreement, the WHO will gather key information from Member and non-Member States and “in partnerships with relevant stakeholders,” which includes the private sector and non-profit actors. While the setup of the Global Supply Chain and Logistics Network includes references to transparency, as will be explained below, these are currently drafted with a “soft” wording, which could lead to important complications in the future.

Lastly, while negotiations for the Annex on the PABS System will continue, several of its essential components have already been adopted by the World Health Assembly. The main text of the Pandemic Agreement now enshrines a minimum of 10% of real-time production of pandemic-related health goods, which should be donated to the WHO. Likewise, any future PABS System will have a direct reference to declarations of a public health emergency of international concern under the IHR (2005). While more detailed components of PABS remain to be negotiated, the elements that have already been adopted touch upon critical private economic interests^17^ that will most likely be present in future pandemics.

## Critical Issues and Missed Opportunities

While the optimism expressed elsewhere is not unjustified, it should nevertheless be tempered with several critical questions about the future of the Pandemic Agreement. Other commentators have expressed skepticism on whether the goals of the Pandemic Agreement are realistic, particularly in the wake of pervasive, preexisting sovereignty considerations.^18^ These criticisms remain, as the political contestation of the Pandemic Agreement is far from over. At the same time, the following critical remarks should be read against the backdrop of the inevitable tradeoffs that are the natural result of a Member State-led process, in which delegations with diverging views negotiate the wording of multiple provisions.

First and foremost, as mentioned above, the entire fate of the Pandemic Agreement will explicitly depend upon the successful conclusion of the PABS Annex. There is, therefore, a real and present risk of a future stalemate between delegations that will impede the finalization of the Pandemic Agreement. While, indeed, some of the key components have already been agreed upon, the highly contested nature of the PABS system looms large. The devil does lie in the details, after all, and a structure failing to guarantee the effective distribution of pandemic-related goods will undermine any lofty goals the agreement might pursue. To complicate things further, the continuing absence of the United States of America will hamper the prospects of an effective PABS System, considering that US corporations have developed the largest volume of new vaccines (up to two-thirds) in past decades.^19^

Closely related to equity considerations under the PABS System, the wording of Article 11 of the Pandemic Agreement on transfer of technology posed a major challenge in the final weeks of negotiations. Stronger commitments will be needed to compel technology-holders — be they private companies, governments, or non-profit institutions — to share knowledge, skills, and expertise with other institutions abroad, especially those in developing countries.^20^ The emphasis on mutually agreed terms as a *sine qua non* condition for this technology transfer will scale down the possibility to gradually increase research and development and manufacturing capacities across countries and regions. At the same time, reference in article 11 to the flexibilities available to states under the World Trade Organization’s Agreement on Trade-Related Aspects of Intellectual Property Rights (TRIPS) reiterates the possibility to use emergency powers for both compulsory licensing and transfer of technology in case of noncooperation by patent holders. While not all types of skills and expertise may be transferred without consent, material technology susceptible of reverse engineering can indeed be subjected to such measures.

Additionally, the gradual watering down of important normative considerations during negotiations on the Pandemic Agreement points to missed opportunities. First, qualifying phrases like “subject to available resources,” “taking into account national circumstances,” and “in accordance with national and/or domestic law” could be used by some states as an excuse to temper the implementation of different provisions of the Pandemic Agreement. A notable example has to do with transparency obligations in the case of research and development and the procurement of pandemic-related health products financed by governments. Past drafts of the Pandemic Agreement did not have such qualifiers,^21^ but rather, for instance, had a direct obligation to publicize information related to both public financing of research and development by the private sector, and to legal decisions regarding research grants and the public procurement of pandemic-related health goods. These issues will be decisive for the proper operation of the Global Supply Chain and Logistics Network, and yet there is currently much leeway for states on whether and how to disclose that information to the WHO or the public at large.

On a similar note, the text of the Pandemic Agreement adopted by the World Health Assembly has a very superficial reference to human rights. Despite expectations for human rights remaining at the core of this field of global health law,^22^ these expectations were not realized in the final Pandemic Agreement. In the current geopolitical context, amid rising state backlash against human rights, the Pandemic Agreement offered a key opportunity to counter those trends under a rights-based approach to pandemic prevention, preparedness, and response.^23^ Doubling down on human rights would have helped counter the growing illiberal contestations of the multilateral system under WHO governance.

Yet the announced WHO withdrawals from the United States and Argentina — beyond questions of their legality — and the ensuing dramatic cutbacks to the WHO’s budget will result in the reduction of WHO personnel and raise major questions about the ability of the organization to fulfill its duties under the Pandemic Agreement. The WHO is meant to have key roles that will guarantee the operability of a future PABS system, the Global Supply Chain Logistics Network, and both the Coordinating Financial Mechanism to be shared between the Pandemic Agreement^24^ and the International Health Regulations (2005).^25^ Should the WHO aim to handle the stewardship of the Pandemic Agreement, if and when it enters into force, its ongoing financial challenges threaten to undermine these prospects.

Moreover, it is worth noting how the procedure for approving the resolution that formally adopted the Pandemic Agreement in the World Health Assembly was not without obstacles. As enshrined in article 60 of the Constitution of the WHO, resolutions adopting binding agreements under article 19 of the WHO Constitution can be approved by a two-thirds majority vote of Member States present and voting at the World Health Assembly. Consensus, in turn, is a decision-making practice by the WHO and several other international organizations, dating back to a particular historical context and meant to ensure that Member States are politically on-board with resolutions, decisions, and other acts, increasing the likelihood of their effective implementation.^26^ The adoption of the main text of the Pandemic Agreement, however, was made through a vote after a request by the delegation of Slovakia — whose current head of government has been particularly acrimonious towards the WHO, the 2024 amendments to the IHR (2005), and the Pandemic Agreement.^27^ In the end, with 124 Member States voting in favor, none against and 11 abstentions, the resulting vote shows the confidence and overwhelming, if not unanimous, support for these new global health law reforms. Perhaps it is time to revisit the custom of resorting to consensus as the end goal of decision-making in international organizations.

## Conclusions

The next phase in the negotiations for a Pandemic Agreement will pose a major test to the capacity of WHO Member States to navigate the geopolitical challenges facing global health law and governance. While the pre-existing liberal order and the role international law played in it was substantially flawed, the emerging leadership vacuum does not offer much refuge or solace for a rules-based coordination for pandemic prevention, preparedness, and response.

The adoption of the Pandemic Agreement is a much-needed win for the WHO, demonstrating that the World Health Assembly is still a functional political forum for reaching understandings on difficult legal questions under global health law. However, the current juncture of the adopted main text of the Pandemic Agreement at the May 2025 World Health Assembly is not a conclusion, but rather a major milestone towards a rules-based system for pandemic prevention, preparedness, and response. Special mention should be made of the roadblocks that delegations at the WHO had to overcome, including external factors such as hostile voices in the media, open rejection of negotiations by political leaders, and occasional pushbacks from the private sector. Sleepless nights and last-minute deals remain ahead because of the pending PABS Annex. Meanwhile, legal scholarship can play a role through both analytical and critical studies, pointing out the potentials and perils of different features of a PABS system and different approaches to implementation. Delegations and stakeholders at the WHO and beyond would benefit from paying heed to those studies, as the task ahead will not be a simple one.
